# Substituting bouts of sedentary behavior with physical activity: adopting positive lifestyle choices in people with a history of cancer

**DOI:** 10.1007/s10552-022-01592-9

**Published:** 2022-06-14

**Authors:** Lee Ingle, Samantha Ruilova, Yunsung Cui, Vanessa DeClercq, Ellen Sweeney, Zhijie Michael Yu, Cynthia C. Forbes

**Affiliations:** 1 Department of Sport, Health & Exercise Science, Faculty of Health Sciences, University of Hull, Hull, UK; 2 Atlantic PATH, Faculty of Medicine, Dalhousie University, Halifax, Canada; 3 Department of Pharmacology, Faculty of Medicine, Dalhousie University, Halifax, Canada; 4 Yorkshire Cancer Research Career Development Fellow, Wolfson Palliative Care Research Centre, Hull York Medical School, University of Hull, Hull, UK

**Keywords:** Isotemporal substitution, Physical activity, Sedentary behavior, Cardiometabolic risk, Oncology

## Abstract

**Purpose:**

To determine in people with a history of cancer, whether substituting sitting time with other daily activities (i.e., sleeping, walking, moderate and vigorous physical activity) was associated with changes in waist circumference (WC), an important surrogate marker of cardiometabolic risk.

**Methods:**

Cross-sectional analyses from the Atlantic Partnership for Tomorrow’s Health (Atlantic PATH) cohort was conducted using isotemporal substitution models to explore the associations of substituting sedentary time, physical activity behavior (International Physical Activity Questionnaire), or sleep (Pittsburgh Sleep Quality Index) with changes in WC. Analyses were conducted using sex-specific WC classifications.

**Results:**

In 3,684 people with a history of cancer [mean age (SD) 58.2 (7.3) years; BMI 28.9 (5.2) kg m^−2^; 71% female], reallocating 10 min of sleep or sedentary time for 10 min of walking was associated with lower WC in women (*p* < 0.01). In men, PA intensity appeared to be more strongly associated with a reduced WC. Replacing 10 min of sedentary time with 10 min of moderate or vigorous PA and replacing 10 min of sleep with moderate PA were associated with a significantly reduced WC (*p* < 0.001). The largest effect was when 10 min of moderate PA was replaced with vigorous PA, a reduction in WC (*p* < 0.01) was evident.

**Conclusion:**

For people with a history of cancer, adopting small but positive changes in lifestyle behaviors could help reduce WC and potentially offset negative health-related outcomes associated with higher WC. Further research is required to examine whether such an intervention may be acceptable and manageable among this population.

## Introduction

Overweight and obesity is the second biggest cause of cancer after smoking culminating in 6% of cancer cases, and is associated with at least 13 independent cancer types in the United Kingdom (UK) [1]. Overweight and obesity are often classified by body mass index (BMI); however, BMI provides limited information regarding the distribution of body fat, nor does it differentiate between muscle and fat mass [2]. A measure of central obesity, waist circumference (WC) provides important information on cardiometabolic risk as it is positively associated with metabolic syndrome in conjunction with several other parameters including raised triglycerides, low levels of high density lipoprotein, hypertension, and elevated fasting glucose levels [3]. In the UK, males and females are classified as being at high risk of cardiometabolic disease if WC > 102 cm and > 88 cm, respectively [4]. Studies have shown a higher incidence of cancer in individuals with a higher WC, thus a higher WC may represent a simple and useful predictor of increased cancer risk [5]. Among those with a cancer diagnosis, having excess weight has been related to poorer quality of life outcomes compared to healthy weight survivors [6, 7], reduced treatment effectiveness [8, 9], more post-surgical complications [10–12], and potentially increased risk of recurrence [13–16], second primary cancers [17], and early mortality [15–18].

Sleep, sedentary time, and physical activity (PA) are daily behaviors which are interdependent, an increase in time in one of these behaviors reduces time spent in one of the others [19]. Among cancer survivors, sleep problems are consistently reported as one of the most burdensome symptoms with insufficient sleep duration and quality reported 11–32% more often among cancer survivors compared to controls without cancer [20, 21]. Accelerometery studies have indicated that sitting time makes up a significant portion of waking hours, often displacing light intensity PA in people with cancer [22]. A recent analysis of pooled accelerometry data found that cancer survivors spend 3% of their daily time in moderate-to-vigorous PA (MVPA), and are sedentary for 66% of their waking hours [23]. However, among those with obesity, significantly more sedentary time and less time in MVPA was observed [23].

Increasing weekly habitual PA levels to ≥ 150 min of moderate or ≥ 75 min of vigorous PA contributes to a number of positive health benefits including a reduction in body fat and WC [24]. Further, among people with a cancer diagnosis, meeting PA guidelines has been strongly associated with better physical function, health-related quality of life, and lower levels of fatigue, anxiety, and depression, than those not meeting guidelines [25–27]. A small to moderate positive association between higher levels of PA and increased total sleep time and sleep quality has been reported; however, this evidence is equivocal [26, 27].

The UK alongside other countries now combine PA guidelines with recommendations for reducing time spent undertaking sedentary behavior [28–30]. Sedentary behavior is defined as “any waking behaviour characterized by an energy expenditure ≤ 1.5 metabolic equivalents (METs), while in a sitting, reclining or lying posture” [31]. Recent evidence indicates that individuals who spend greater time adopting a sedentary lifestyle, such as habitual prolonged desk sitting of < 1.5 metabolic equivalents, are at an increased risk of cancer and cardiovascular disease [32, 33]. Compared with uninterrupted sitting time, adding bouts of light or moderate intensity PA may reduce the risk of cardiometabolic disease [34–37], and certain cancers [25].

Traditionally, PA recommendations focused on continuous moderate-to-vigorous PA. By the mid‐1980s, evidence showed that PA completed in shorter, 10‐minute blocks also provided significant health benefits [24]. Consequently, some self‐reported questionnaires were used to obtain PA data for population surveillance in 10‐min bouts, such as the Active Australia Survey [38]. Recommendations to add PA in small manageable bouts such as 10-min blocks may be particularly useful, acceptable, and realistic to cancer survivors who generally have sedentary lifestyles [39].

We used isotemporal substitution modeling analyses to determine the impact of adopting positive behavioral choices and their association with WC in a large population of people living with and beyond cancer. For example, substituting short bouts of daily sedentary (sitting) time for light, moderate, or vigorous PA, or sleep. This method has been used to examine associations in health outcomes across young populations, adults and the elderly, and specific clinical populations [40]. This technique has been used in people with breast cancer to determine associations in quality of life [41], WC and BMI [19], cancer-related cognitive impairment [42], and cancer recurrence biomarkers [43] among people with colorectal cancer to look at associations in health-related quality of life [44], and among people with non-Hodgkin lymphoma to explore associations in fatigue and quality of life [45]. However, in some cases, only waking time activities were included [42, 43]. The purpose of the study is to determine the associations between WC and reallocating sedentary time to sleep, walking or MVPA in people with a history of cancer. We hypothesized that reallocating 10 min per day of sedentary time or sleep with 10 min per day of any intensity of PA would be associated with a reduced WC.

## Methods

Ethical approval for secondary data analysis was provided by the Department of Sport, Health & Exercise Ethics Committee at the University of Hull (Reference number: 1920130). Atlantic Partnership for Tomorrow’s Health (Atlantic PATH), a regional cohort (*n* = 31,173) of the Canadian Partnership for Tomorrow’s Health (CanPath), provided approval to access data [46]. CanPath is a national longitudinal cohort study with more than 333,000 participants examining the influence of health, lifestyle, environment, and behavior on the development of chronic disease [47]. The detailed methods of Atlantic PATH have been previously published [46]. Briefly, more than 31,000 people aged between 35 and 69 years were recruited between 2009 and 2015 across four Eastern Canadian provinces (Nova Scotia, New Brunswick, Newfoundland & Labrador, and Prince Edward Island). Participants gave informed consent and completed a set of questionnaires that assessed sociodemographic characteristics, current health status, history of disease (personal and family), and lifestyle behaviors (i.e., sleep, physical activity, diet, smoking, and alcohol consumption). Copies of questionnaires with all items can be viewed at https://www.atlanticpath.ca/index.php/are-you-a-researcher/. Body composition measures (stature, body mass, % fat mass) were collected at assessments centers or via mobile clinics held across the provinces.

This analysis included 3,684 participants aged 35–69 who indicated they had a previous cancer diagnosis and who completed detailed questionnaires including information on medical history, health behaviors, and completed the physical measurements.

### Sociodemographic and behavioral factors

Participants reported age, sex, education levels, employment, marital status, household income, smoking status, weekly alcohol consumption, self-reported sleep duration, and fruit and vegetable consumption. In addition to cancer type and treatment received, participants also reported any doctor-diagnosed comorbidities (e.g., heart disease, diabetes, etc.) and their self-rated health on a commonly used 5-point Likert scale (1 = poor, 5 = excellent) [48, 49].

### Self-reported behaviors

#### Physical activity

Total daily habitual PA was assessed using the short- and long-form of the International Physical Activity Questionnaire (IPAQ) [50]. All participants were offered the short-form IPAQ as part of the Core Questionnaire common to CanPATH; however, 68% of Atlantic Canadian participants also completed a supplemental questionnaire that included the long-form. The long-form IPAQ specifies domains of activity including occupational, transportation, household/domestics activities, and leisure-time activity. The short-form focuses on general information related to each intensity category (walking, moderate, or vigorous intensity). If participants completed both, we opted to include long-form data. Participants reported the average amount of time spent per week engaged in walking, moderate, or vigorous activities by indicating how many times per week they engaged in each activity, and the mean duration of each session. Calculated categorical PA scores as defined by IPAQ were found to be highly skewed [51]. Therefore, data-driven tertiles for total metabolic expenditure (MET-minutes per week) were calculated for both the short- and long-form tools separately to determine whether participants engaged in low, moderate, or high levels of PA (MET-minutes per week). These tertiles were used for descriptive purposes; isotemporal substitution analyses used calculated minutes for each category.

### Sedentary behavior

The short- and long-form IPAQ was also used to assess time spent sitting. In the long-form IPAQ, participants were asked to indicate, over the past week, how many days and for how long they spent traveling in a motor vehicle (converted into average minutes per day) and how much time in total (excluding time in a motor vehicle) they spent sitting on a weekday and on a weekend day. Short-form IPAQ asked participants to indicate how much time on average they spent sitting on a weekday and on a weekend day.

#### Sleep patterns

Participants reported their sleep duration and quality by answering the following survey questions. Firstly, total daily/nightly time asleep by reporting in hours and minutes (e.g., 7 h, 30 min) “*On average, how many hours per day/night do you usually sleep, including naps? A day refers to a 24-h period. Please think of the total amount of unbroken sleep.*” Participants also reported sleep quality by answering “*How often do you have trouble going to sleep or staying asleep?*” on a 5-point Likert scale with options from “*never*” to “*all the time.*” Sleep time was converted into categories for descriptive purposes.

#### Physical measurements

Body weight was measured at an assessment center or mobile clinic using a Tanita bioelectrical impedance device (Tanita BC-418; Tanita Corporation of America, Arlington Heights, Illinois, USA). Stature was measured using a stadiometer (Seca, Brooklyn, NY, USA) and combined with body mass was used to calculate BMI (kg·m^−2^). Waist and hip circumference (cm) were measured using a Lufkin steel tape (Ohio, USA) by staff at assessment centers or self-reported in home assessment packages. Two-thirds of the AtlanticPATH sample provided measures at assessment clinics, and self-reported stature, body mass, and waist circumference were also collected and used in cases where clinic measures were not available.

### Statistical analysis

For descriptive purposes, participants were grouped based on WC; females with a WC < 80 cm and males with a WC between < 94 cm were classified as low risk. Females with a WC between 80 and 88 cm, and males with a WC 94–102 cm were classified as high risk. Females with a WC > 88 cm and males with a WC > 102 cm were classified as very high risk [4]. Differences in participant characteristics based on WC-associated risk categories were calculated using analysis of variance (ANOVA) for continuous variables and the Chi-square test for categorical variables. Descriptive data were reported as mean (95% CI) or percentages. Data analysis was performed using SPSS version 24 (IBM, NY, USA). Missing data were treated by multiple imputation in five imputed datasets.

The isotemporal substitution method has been described in-depth elsewhere [19]. We converted sitting time (sedentary behavior), sleeping time, walking, moderate PA, and vigorous PA into daily units of 10 min. We also created a “total time” variable, which was the sum of these activities and converted it to a 10-min unit variable. Linear regression models were used to estimate the association between key activity variables (sleep duration, sitting time, walking, moderate PA, and vigorous PA) and the original continuous WC outcome. All models were run separately for men and women due to differences in WC and health risk. Variables considered as covariates were age, marital status, education level, employment status, household income, ethnicity, alcohol consumption, smoking status, self-rated health, number of comorbidities, BMI, and daily fruit and vegetable consumption. Firstly, single effects models were run to determine covariate associations in each activity category (e.g., covariate associations between WC and sleep only, excluding walking, sitting, moderate, and vigorous). Secondly, partition models were run with all activity outcome variables (therefore mutually adjusted) and covariates trending toward significance *(p* ≤ 0*.*10) in the single effects models to determine associations with WC. Variables with trends toward significance (*p* ≤ 0.10) at this stage were included in the isotemporal models. No serious autocorrelation for partition models was observed (Durbin-Watson score = 2.029), and variance inflation factors for each activity variable were below 1.3 indicating a very low risk of multicollinearity. All other linear assumptions were met.

Three isotemporal models were included in the final analysis and each run separately for males and females; unadjusted with no covariates (model 0); model 1 adjusted for sociodemographic factors only (males: age, education; females: marital status, employment, household income); and model 2 further adjusted for sociodemographic plus behavioral factors (males: model 1 plus self-rated health, BMI; females: model 1 plus smoking status, self-rated health, comorbidities, BMI). The B coefficient from the regression analysis is an estimate of the pooled mean effect on the outcome (WC) of reallocating 10 min of an omitted activity (sleep duration, sitting/sedentary time, walking, moderate PA, or vigorous PA) with 10 min of each included activity, while other activities are kept constant. Activity reallocation estimates change the outcome at the population level, not the individual level, and outputs are associative rather than causal reallocations of time between different key activity variables and the outcome [52]. An alpha level of *p* < 0.05 was considered significantly meaningful.

## Results

We identified a cohort of 3,684 participants [mean age (SD) 57.2 (8.1) years; BMI 28.4 (5.9) kg m^−2^; 71% female] living with and beyond cancer. The 11 most prevalent types of cancer are reported in Table 1, less prevalent types were classified as “other” for ease of reporting. Sixty percent of the sample indicated they had received some treatment for their cancer. Of those that specified treatment types, most indicated having just one type of treatment (62%). Females classified at low risk based on WC had a significantly lower BMI than females at high risk (*p* < 0.001). Likewise, females at very high risk had a BMI significantly higher than females at high risk (*p* < 0.001). This pattern was also evident in males (*p* < 0.001). In the low-risk category, 27% of females and 49% of males had no co-morbidities. Detailed characteristics and differences based on WC risk categories are found in Table 1.

**Table 1:** Baseline clinical characteristics of participants living with and beyond cancer

Variables	Waist circumference					
	Low risk		High risk		Very high risk	
	Male < 94 cm	Female < 80 cm	Male 94-102 cm	Female 80-88 cm	Male > 102 cm	Female > 88 cm
	*N* = 413	*N* = 411	*N* = 256	*N* = 498	*N* = 267	*N* = 1351
Age (years)^b^	58.8 (58.0–59.5)^c^	55.7 (54.9–56.4)	60.3 (59.5–61.1)^c^	56.6 (55.9–57.3)	60.6 (59.8–61.4)^c^	56.5 (56.1–56.7)
Rurality^a^						
Urban	45.6	18.3	26.8	22.9	27.6	58.8
Rural	40.9	17.4	28.8	20.7	30.3	61.9
Marital status^a^						
Married/common law	44.0	17.7	26.8	21.9	29.2	60.3
Not Married	44.9	19.5	31.8	22.4	23.4	58.1
Education level^a^						
High School or lower	42.1	17.6	24.3	21.8	33.7	60.6
Postsecondary or higher	44.8	18.4	28.4	22.1	26.8	59.5
Employment status^a^						
Employed full-time	46.3	17.4	24.8	20.5	29.0	62.1
Employed part-time	43.1	22.0	30.6	25.4	26.4	52.7
Retired	40.0	17.2	29.1	24.2	30.9	58.5
Not employed	49.0	19.6	30.8	20.1	20.2	60.3
Household Income^a^						
< $75,000 per year	43.4	16.9	28.3	22.7	28.3	60.5
≥ $75,000 per year	44.1	20.1	28.6	20.6	27.3	59.3
Ethnicity^a^						
White ethnicity	44.0	18.3	27.6	22.1	28.4	59.6
Non-white ethnicity	44.9	16.9	22.4	19.5	32.7	63.6
Waist circumference (cm) ^b^	83.6 (82.7–84.6)^c^	71.1 (70.1–72.2)^d^	98.2 (97.9–98.5)^c^	84.1 (83.9–84.3)^d^	112.2 (111.1–113.3)^c^	102.8 (102.1–103.4)^d^
BMI (kg∙m^−2^)^b^	27.3 (26.7–27.8)^c^	25.1 (24.5–25.6)^d^	28.0 (27.3–28.6)^c^	26.6 (26.2–27.1)^d^	30.6 (30.0–31.2)^c^	29.9 (29.6–30.2)^d^
Co-morbidities^a^						
None	48.7^c^	27.2^d^	33.6	25.4	17.6^c^	47.4^d^
One	46.6	20.1	26.4	23.6	27.1	56.3
Two	44.4	18.1	27.8	20.2	27.8	61.7
Three	37.7	14.7	26.3	20.2	35.9	65.1
Four (or more)	41.8	12.6^d^	23.6	22.2	34.5	65.3^d^
Activity Behavior						
Walking (min per day)^b^	58.0 (52.3–63.6)	64.7 (58.8–70.5)^d^	65.2 (58.0–72.4)	58.9 (53.8–64.0)	55.3 (48.4–62.3)	51.3 (48.4–54.2)^d^
Moderate PA (min per day)^b^	68.7 (62.2–75.2)^c^	79.4 (72.8–86.0)	82.0 (73.3–90.6)^c^	76.5 (70.5–82.5)	79.5 (71.0–88.0)	73.4 (69.8–77.0)
Vigorous PA (min per day)^b^	37.8 (32.6–43.0)	26.8 (22.5–31.1)	36.8 (30.3–43.4)	23.1 (19.5–24.3)	31.6 (25.6–37.7)	22.0 (19.7–24.3)
Sitting time (hours per day)^b^	5.9 (5.6–6.2)^c^	5.5 (5.2–5.8)^d^	6.2 (5.8–6.5)^c^	5.4 (5.1–5.6)^d^	6.9 (6.4–7.4)^c^	6.0 (5.8–6.2)^d^
Vegetable servings per day^b^	2.1 (2.0–2.3)	2.8 (2.7–3.0)	2.3 (2.1–2.5)	2.7 (2.6–2.9)	2.2 (2.0–2.4)	2.7 (2.7–2.8)
Fruit servings per day^b^	1.8 (1.7–1.9)	2.3 (2.1–2.4)	1.8 (1.6–2.1)	2.3 (2.2–2.5)	1.7 (1.6–1.9)	2.2 (2.2–2.3)
Hours sleep per day^b^	7.3 (7.1–7.4)	7.2 (7.0–7.3)	7.3 (7.1–7.4)	7.2 (7.1–7.3)	7.3 (7.1–7.4)	7.2 (7.2–7.3)
Alcohol consumption^a^						
Never drink	45.7	18.3	21.3	20.0	33.0	61.7
< 1 drink a week	42.9	15.8^d^	27.3	20.3^d^	29.8	63.9^d^
1–3 drinks per week	43.5	21.7^d^	29.7	22.2	26.8	56.1^d^
≥ 4 drink per week	44.0	20.8	28.2	27.3^d^	27.8	51.9^d^
Smoking status^a^						
Never smoked	45.1	20.9^d^	28.3	22.6	26.7	56.5^d^
Ex-smoker	43.2	16.3^d^	26.2	20.7	30.6	63.1^d^
Current smoker	45.9	14.3	29.4	25.5	24.7	60.2
Self-rated health^a^						
Poor/Fair	40.0	12.8	24.3	15.4	35.7	71.8^d^
Good	39.2	14.3^d^	29.7	22.3	31.2	63.3
Very good/excellent	49.1^c^	22.0	26.8	23.6	24.1^c^	54.4^d^
Cancer type^a^						
Breast	75.0	15.3	25.0	22.3	0.0	62.3
Brain	75.0	28.6	0.0	14.3	25.0	57.1
Blood	45.6	20.6	22.1	23.8	32.4	55.6
Colorectal	40.7	22.5	27.1	18.3	32.2	59.2
Gynaecological	–	15.7	–	17.1	–	67.2
Head & Neck	66.7	20.5	19.0	16.4	14.3	63.0
Respiratory Tract	80.0	12.5	20.0	25.0	0.0	62.5
Gastrointestinal	80.0	36.4	6.7	18.2	13.3	45.5
Genitourinary	40.0	8.8	35.6	32.4	24.4	58.8
Prostate	44.1	–	26.5	–	29.4	–
Skin	44.1	16.5	28.6	21.9	27.3	61.6
Other	45.0	11.7	10.0	21.7	45.0	66.7
Ever had treatment^a^	47.4	16.6	25.1	21.1	27.5	62.3
Chemotherapy^a^	49.5	13.9	20.6	23.9	29.9	62.2
Radiation therapy^a^	45.5	16.7	22.4	21.3	32.1	62.0
Surgery^a^	47.6	16.9	26.5	21.2	25.9	61.9

^a^Values expressed as a (%)

^b^Values expressed as mean (95% CI)

^c^Differences between male groups

^d^Differences between female groups

### Single effects and partition models

In the single effects model, increased sitting time was the only activity associated with an increase in WC in men (*p* < 0.001). In women, increased moderate PA (*p* = 0.004) and increased walking time (*p* < 0.001) were associated with decreased WC, while increased sitting time (*p* < 0.001) and increased sleep (*p* = 0.031) were associated with increases in WC. After all activities were mutually adjusted, increased sitting time (*p* < 0.001) and increased moderate PA (*p* < 0.001) were associated with increased WC in men. In women, increased walking time (*p* = 0.007) was associated with decreased WC, whereas increased sitting time (*p* < 0.001) was associated with increased WC (Table 2).

**Table 2:** Unadjusted single effects and partition models for men and women

	Difference in waist circumference (cm)			
	Men (*n* = 990)		Women (*n* = 2353)	
	Single effects^a^	Partition^b^	Single effects^a^	Partition^b^
Behavior (10 min per day)	Β(95% CI)	Β(95% CI)	Β(95% CI)	Β(95% CI)
Vigorous PA	− 0.178 (− 0.386 to 0.030)	− 0.176 (− 0.395 to 0.043)	− 0.177 (− 0.372 to 0.018)	− 0.030 (− 0.227 to 0.167)
Moderate PA	0.121 (− 0.009 to 0.252)	0.249 (0.101 to 0.397)*	− 0.150 (− 0.250 to − 0.049)*	− 0.014 (− 0.120 to 0.092)
Walking	− 0.075 (− 0.233 to 0.082)	− 0.067 (− 0.244 to 0.110)	− 0.302 (− 0.434 to − 0.171)*	− 0.206 (− 0.353 to − 0.058)*
Sitting	0.108 (0.055 to 0.149)*	0.115 (0.059 to 0.170)*	0.127 (0.089 to 0.164)*	0.108 (0.066 to 0.150)*
Sleep	− 0.022 (− 0.141 to 0.098)	− 0.029 (− 0.148 to 0.091)	0.091 (0.009 to 0.174)*	0.076 (− 0.004 to 0.157)

^a^Unadjusted model

^b^Adjusted for other activity behaviors

*p* < 0.05

### Isotemporal models

**Fig. 1:**
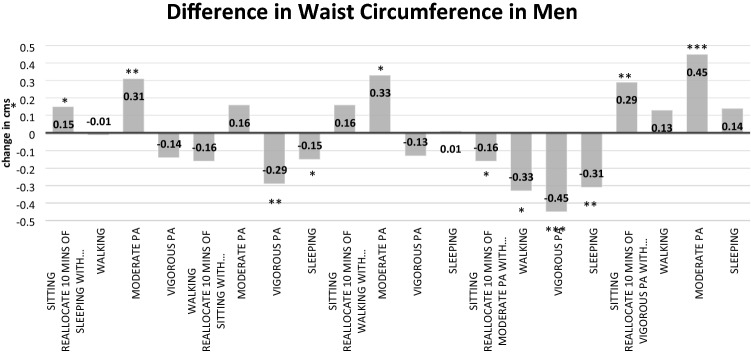
Changes in waist circumference (cm) in men when substituting 10 min of one behaviour for another (fully adjusted model). *Significant association *p* < 0.05; **significant association *p* < 0.01; ***significant association *p* < 0.001

**Fig. 2:**
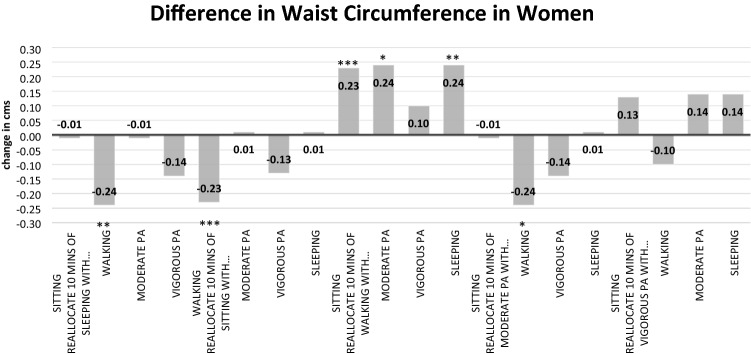
Changes in waist circumference (cm) in women when substituting 10 min of one behavior for another (fully adjusted model). *Significant association *p* < 0.05; **significant association *p* < 0.01; ***significant association *p* < 0.001

Full details of the three isotemporal substitution models for males are found in Table 3. In the fully adjusted model, a significant decrease in WC was observed when replacing 10 min of moderate PA with 10 min of sleeping time [Δ − 0.31 (− 0.50 to − 0.12) cm; *p* = 0.002], 10 min of sitting time [Δ − 0.16 (− 0.32 to − 0.01) cm; *p* = 0.043], 10 min of walking [Δ − 0.33 (− 0.61 to − 0.04) cm; *p* = 0.026], or 10 min of vigorous PA [Δ − 0.45 (− 0.72 to − 0.19) cm; *p* < 0.001]. Details of all isotemporal substitution models for females are available in Table 4. Reallocating 10 min of sleeping time [Δ − 0.24 (− 0.39 to − 0.09) cm; *p* < 0.001], sitting/sedentary time [Δ − 0.23 (− 0.34 to − 0.11) cm; *p* < 0.001], or moderate PA [Δ − 0.24 (− 0.43 to − 0.05) cm; *p* = 0.014] with 10 min of walking was associated with a significantly lower WC. All other associations in males and females were non-significant. Changes in WC (fully adjusted models) for males and females based on activity behavior substitutions are presented in Figs. 1 and 2.

**Table 3:** Isotemporal substitution of lifestyle-related activities on waist circumference in males [B (95% CI)]

Reallocating 10 min of…	With 10 min of…	Impact on waist circumference (Δ cm)		
		Unadjusted	Model 1^a^	Model 2^b^
*Activity*				
Sleeping	Walking	− 0.01 (− 0.22 to − 0.20)	− 0.01 (− 0.22 to − 0.20)	− 0.01 (− 0.22 to − 0.19)
Sleeping	Moderate PA	0.31 (0.12 to 0.50)**	0.30 (0.11 to 0.49)**	0.31 (0.12 to 0.50)**
Sleeping	Vigorous PA	− 0.16 (− 0.38 to 0.07)	− 0.14 (− 0.36 to 0.08)	− 0.14 (− 0.36 to 0.07)
Sitting/sedentary	Walking	− 0.17 (− 0.36 to 0.03)	− 0.18 (− 0.37 to 0.02)	− 0.16 (− 0.35 to 0.03)
Sitting/sedentary	Moderate PA	0.15 (− 0.01 to 0.31)	0.14 (− 0.02 to 0.30)	0.16 (0.01 to 0.32)*
Sitting/sedentary	Vigorous PA	− 0.31 (− 0.51 to − 0.12)**	− 0.30 (− 0.50 to − 0.11)**	− 0.29 (− 0.48 to − 0.10)**
Sitting/sedentary	Sleep	− 0.15 (− 0.28 to − 0.03)*	− 0.16 (− 0.29 to − 0.04)**	− 0.15 (− 0.27 to − 0.03)*
Walking	Moderate PA	0.32 (0.03 to 0.60)*	0.32 (0.03 to 0.60)*	0.33 (0.04 to 0.61)*
Walking	Vigorous PA	− 0.14 (− 0.44 to 0.15)	− 0.13 (− 0.42 to 0.17)	− 0.13 (− 0.41 to 0.16)
Moderate PA	Vigorous PA	− 0.46 (− 0.73 to 0.19)***	− 0.44 (− 0.71 to 0.17)**	− 0.45 (− 0.72 to − 0.19)***

*Significant association *p* < 0.05

**Significant association *p* < 0.01

***Significant association *p* < 0.001

^a^Model 1 adjusted for age, education

^b^Model 2 adjusted for age, education, perceived general health, body mass index

**Table 4:** Isotemporal substitution of lifestyle-related activities on waist circumference in females [B (95% CI)]

Reallocating 10 min of…	With 10 min of…	Impact on waist circumference (Δ cm)		
		Unadjusted	Model 1^a^	Model 2^b^
*Activity*				
Sleeping	Walking	− 0.26 (− 0.42 to − 0.09)**	− 0.27 (− 0.43 to − 0.10)**	− 0.24 (− 0.39 to − 0.09)**
Sleeping	Moderate PA	− 0.07 (− 0.22 to 0.07)	− 0.09 (− 0.24 to 0.06)	− 0.01 (− 0.14 to 0.13)
Sleeping	Vigorous PA	− 0.10 (− 0.32 to 0.12)	− 0.11 (− 0.33 to 0.11)	− 0.14 (− 0.35 to 0.06)
Sitting/sedentary	Walking	− 0.29 (− 0.43 to − 0.15)***	− 0.29 (− 0.44 to − 0.15)***	− 0.23 (− 0.34 to − 0.11)***
Sitting/sedentary	Moderate PA	− 0.11 (− 0.23 to 0.01)	− 0.12 (− 0.24 to 0.00)	− 0.01 (− 0.10 to 0.11)
Sitting/sedentary	Vigorous PA	− 0.14 (− 0.35 to 0.08)	− 0.14 (− 0.35 to 0.08)	− 0.13 (− 0.33 to 0.06)
Sitting/sedentary	Sleep	− 0.03 (− 0.13 to 0.07)	− 0.03 (− 0.13 to 0.07)	0.13 (− 0.08 to 0.11)
Walking	Moderate PA	0.18 (− 0.03 to 0.39)	0.18 (− 0.03 to 0.39)	0.24 (0.05 to 0.43)*
Walking	Vigorous PA	0.16 (− 0.10 to 0.42)	0.16 (− 0.10 to 0.42)	0.10 (− 0.14 to 0.34)
Moderate PA	Vigorous PA	− 0.03 (− 0.29 to 0.23)	− 0.02 (− 0.28 to 0.24)	− 0.14 (− 0.37 to 0.10)

*Significant association *p* < 0.05

**Significant association *p* < 0.01

***Significant association *p* < 0.001

^a^Model 1 adjusted for marital status, employment, income category

^b^Model 2 adjusted for marital status, employment, income category, smoking status, perceived general health, comorbidities, body mass index

## Discussion

We found in a large sample of men and women with a history of cancer that small positive changes in daily activity behaviors such as replacing 10 min of sedentary behavior with 10 min of PA is associated with a reduced WC. These small bouts can be accrued throughout the day by making positive incidental changes in behaviors. Specifically, in women, we found that reallocating 10 min of sitting/sedentary time or sleeping time with 10 min of walking was associated with a small but significant reduction in WC. Likewise, in men, replacing 10 min of sleeping/sitting/sedentary time with 10 min of moderate or vigorous PA was associated with a small but significant reduction in WC. In men, PA intensity was more strongly associated with a reduced WC. The largest reduction in WC was observed when 10 min of moderate PA was replaced by 10 min of vigorous PA.

Boyle and colleagues [19] investigated the interdependent associations of self-reported sleep, and objectively measured bouts of sedentary time, light PA, and moderate-to-vigorous PA (MVPA) on WC and BMI in 256 breast cancer survivors. The authors reported that reallocating 30 min to MVPA was significantly associated with lower WC when allocated from sleep (− 2.5 cm), prolonged sedentary time (− 2.5 cm), or light PA (− 2.7 cm). The magnitude of the reduction in WC is higher than in our study. Boyle and colleagues [19] included 30-min bouts of MVPA, whereas we included 10-min bouts indicating that there may be a dose-dependent reduction in WC, the magnitude of the reduction in WC is associated with the amount of MVPA undertaken. While their study used objective and well-validated measures to calculate PA (Actigraph accelerometers), the study was limited by a relatively low sample size and focused on a single cancer type, whereas our study is significantly larger and the findings are more generalizable to other cancer diagnoses. The 10-min bouts of PA which we reallocated in our study may be more realistic for sedentary individuals to adopt, at least initially, if a concerted effort is made to adopt a healthier lifestyle.

Other studies using isotemporal substitution modeling have shown the benefits of reallocating sedentary for active behavior for improving aspects of health-related quality of life in 145 patients with lymphoma [53], and fatigue and quality of life in 753 patients with breast cancer [54]. Isotemporal substitution modeling has also been used to identify clinically important minimal thresholds; for example, reallocating ~ 65 min per day of sedentary time to light PA was associated with clinically meaningful improvements in fatigue in 463 kidney cancer survivors [55]. We are unable to provide any clinically meaningful thresholds from our analysis as we have focused on small (10 min) bouts of incidental changes in lifestyle behaviors. If these small changes in lifestyle are maintained or increased over time, we can speculate that this could improve the risk burden in this cohort.

Our study showed that men and women require different strategies to optimize reductions in WC. We found that women may observe greater reductions in WC by engaging in light PA, whereas men required more intensive PA to yield more significant reductions. It is not clear why this would be the case. However, it is clear that overweight and obesity worsen cancer outcomes; for example, women with a BMI > 40 kg m^−2^ have a 60% higher risk of dying from any cancer than women with normal weight [56]. In 2014, the American Society for Clinical Oncology released a call to action for obesity counseling and management by oncology service providers [57]. However, weight management strategies are not routinely integrated into cancer care services, and our findings reinforce this message.

The role of WC as a surrogate marker of cardiometabolic risk in our study was important. Our findings show that small positive changes in lifestyle behaviors will lead to small but positive reductions in WC. Over prolonged periods, this could mean that individual cardiometabolic risk profiles are improved and individuals could be reclassified into lower risk categories. Waist circumference is a proxy measure of central adiposity [58], and may reflect elevated levels of subcutaneous abdominal and/or visceral fat which impairs insulin control [59]. High levels of adipose tissue may trigger elevated pro-inflammatory cytokine responses such as elevated tumor necrosis factor alpha (TNF-α) and interleukin 6 (IL-6). Both IL-6 and TNF-α are associated with tumor development and metastasis [59], and the inhibition of tumor growth suppressors in people with cancer [60]. Adopting small daily changes in lifestyle behaviors over time could mean that some of these negative physiological outcomes associated with higher levels of WC are reduced.

Successful adoption of MVPA guidelines remains challenging in cancer populations [61]. Only 8% of cancer survivors meet weekly PA recommendations and spend an average of 16 min per day performing MVPA [62]. Our study highlights the value of replacing sedentary behavior with some form of PA for minor reductions in WC in males and females living with and beyond cancer. Initially at least, this may be a more useful and realistic intervention for trying to engage people with a more active lifestyle and replacing sedentary behavior (e.g., increasing leisure-time walking, light housework, and gardening) [41]. Barriers to PA participation in cancer populations include lack of time, competing interests, safety concerns, lack of knowledge, equipment and support, and adequate and relevant training of allied healthcare professionals supporting people with cancer [22–25]. Cancer-specific issues include dealing with fatigue, and the side effects of medication and treatment [26–28]. Initiating changes in lifestyle with lighter PA intensities would be recommended for most people with cancer, and it should be possible to increase the frequency, intensity, and duration of PA over time, depending on, for example, individual levels of ability, mobility, and feelings of fatigue [27].

In people living with and beyond cancer, disrupted sleep can lead to increased risk of morbidity and mortality, and poorer quality of life [63, 64]. Disrupted sleep is associated with a higher risk of cancer-specific mortality, and these risks are markedly exacerbated among people who are sedentary or do insufficient PA to meet guidelines [65]. Huang and colleagues [65] showed in a recent meta-analysis that meeting the lower threshold of the current PA guidelines (600 MET-mins per week) eliminated most of the deleterious associations between disrupted sleep and increased mortality risk. Therefore, our findings that making small but positive changes to lifestyle behaviors, for example, by increasing habitual levels of PA should be encouraged in people living with and beyond cancer.

Since the start of the global pandemic, society has been enormously impacted by COVID-19, with many citizens being required to stay at home to reduce transmission risk. The negative consequences on physical and mental wellbeing have yet to be fully determined. However, a recent report by Di Sebastiano et al. [62] indicated that in 2,338 middle-aged Canadians, MVPA and light PA had significantly declined immediately following the declaration of the COVID-19 pandemic. While MVPA had returned to pre-pandemic levels six weeks later, light PA remained significantly lower than pre-pandemic levels. These significant and sustained declines in incidental light PA should be a public health priority. Our study highlights that replacing 10 min of sedentary behavior with 10 min of incidental light PA, such as walking is associated with a minor reduction in WC which has the potential to improve cardiometabolic risk. While citizens may have been unable to attend gyms or facility-based exercise classes due to COVID-19-related public health restrictions, healthcare practitioners, policymakers, and commissioning groups should be actively promoting and encouraging small positive changes in lifestyle behaviors. Our study provides strong supporting evidence for these incremental increases in incidental light PA within a society where movement has been recently restricted.

Our study is not without its limitations. The majority of measures were self-reported and therefore subject to recall and measurement biases. In order to measure PA, we used a questionnaire which has inherent challenges with individuals being able to accurately recall the amount and intensity of PA they have engaged in over the past seven days. Additionally, when completing self-report surveys like the IPAQ, PA is often overestimated by participants, while sedentary behavior is often underestimated. Finally, the IPAQ inherently omits other light intensity activities by asking about walking only. It is possible the estimations and inherent limitations of the IPAQ may have impacted the strength of associations found in our results. However, the IPAQ does allow for comparing groups within or between countries in large surveillance studies rather than focusing on a participants’ individual risk [59]. A strength of our study is the sample size with > 3500 people living with and beyond cancer included in our analysis, which is much larger than previous investigations using similar methodologies [40–45].

Future studies could statistically investigate increasing the daily duration of positive behavioral choices (e.g., 30 min or 1 h) to see if there is a dose–response relationship. It is also possible that our mixed results for swapping sedentary behavior for some form of PA may have been clouded by using recall questionnaires rather than objective measurements of sedentary time and PA. Accelerometry would have allowed us to capture discrete bouts of MVPA more accurately. Furthermore, inclinometry would have allowed us to accurately quantify sitting time (versus standing time) which is particularly relevant in people living with and beyond cancer who may suffer from chronic fatigue, negatively impacting their health-related QoL. However, using such technology is time consuming and costly especially in large-scale epidemiological studies.

In conclusion, our study highlights that small changes in behavior such as replacing 10 min of sedentary behavior with 10 min of PA are associated with a minor reduction in WC in males and females with a history of cancer. These small bouts can be accrued throughout the day by making incidental changes in behavior and may, over time, result in small but positive improvements in health outcomes. These findings are timely and positive given recent public health restrictions related to COVID-19.

## Data Availability

Data and biosamples from Atlantic PATH are available to researchers through a data access process. Additional information can be obtained by contacting info@atlanticpath.ca.
